# Non-B DNA-informed mutation burden as a marker of treatment response and outcome in cancer

**DOI:** 10.1038/s41416-024-02873-7

**Published:** 2024-10-19

**Authors:** Qi Xu, Jeanne Kowalski

**Affiliations:** 1 Department of Oncology, Dell Medical School, The University of Texas at Austin, Austin, TX USA; 2 Department of Molecular Biosciences, College of Natural Sciences, The University of Texas at Austin, Austin, TX USA

**Keywords:** Cancer, Biomarkers, Oncology, Genomic instability

## Abstract

**Background:**

Genomic instability is crucial in tumorigenesis, with Tumour Mutation Burden (TMB) being a biomarker to indicate therapeutic effectiveness, particularly in immunotherapy. However, TMB is not always a reliable predictor and displays heterogeneity. Non-B DNA, susceptible to mutations, play a significant role in cancer development, indicating their potential merit when combined with mutation for enhanced markers in cancer.

**Methods:**

We assessed mutations and non-B DNA interplay as biomarkers. Our methodology quantifies tumour mutations and their co-localization with non-B DNA, using survival and drug sensitivity assessments for clinical relevance.

**Results:**

We introduce two novel markers, ‘nbTMB’ (non-B-informed tumour mutation burden) and ‘mlTNB’ (mutation-localised tumour non-B burden). In case studies: (1) nbTMB informs on survival heterogeneity among TMB-high patients undergoing immunotherapy whereas TMB is unable to further differentiate; (2) nbTMB informs on altered cisplatin sensitivity among ovarian cancer cell lines whereas TMB is unable to differentiate; and (3) mlTNB informs on survival heterogeneity among early-stage pancreatic cancer progressors in whom other markers of genomic instability fail to differentiate.

**Conclusions:**

These novel markers offer a nuanced approach to enhance our understanding of treatment responses and outcomes in cancer, underscoring the need for a comprehensive exploration of the interplay between non-B and B-DNA features.

## Introduction

Genomic instability, marked by numerous genetic changes due to impaired DNA repair processes, stands as a hallmark of cancer [1]. It plays an important role in contributing to the cancer heterogeneity, therapeutic resistance and association with poor prognosis [2, 3]. Tumour Mutation Burden (TMB) stands as a crucial metric in oncology, quantifying the total mutations within tumour. It serves as a biomarker for the effectiveness of immunotherapies, particularly immune checkpoint inhibitors [4]. A higher TMB often indicates a more favourable response to immunotherapies, providing a vital tool for clinicians in customising therapeutic strategies and anticipating treatment outcomes [5]. However, the use of TMB and its threshold is not uniform across different cancer types, indicating the need for more nuanced exploration.

Within the genomic landscape, non-B DNA structures emerge as notable entities [6–8]. These structures deviate from the conventional B-DNA double helix to form alternative structures [6]. They disrupt the standard processes of DNA replication and transcription, thereby laying the foundation for genetic instability. The propensity of these structures to induce mutations underscores their critical role in cancer initiation and progression [9–13]. Their increased susceptibility to change gives rise to an abundance of population variants linked to non-B DNA motifs and an amplified frequency of somatic mutations at these sites, notably in cancer contexts. Even though numerous variants tied to non-B DNA motifs may not have a profound impact, these motifs play a pivotal role in the genetic diversity of the human genome [14, 15]. As a result, they stand out as primary areas of interest for disease development and genetic discrepancies [15]. It is essential to factor in the importance of non-B DNA motifs for predicting mutation frequencies and evaluating potential disease risks, while developing new biomarkers in the context of cancer.

In this study, we aim to introduce two novel biomarkers: nbTMB (non-B-informed TMB) and mlTNB (mutation-localised tumour non-B DNA burden). These biomarkers are designed to quantify the multifaceted nature of genomic instability by accounting for both tumour mutations and non-B DNA at the sample level. Our goal is to evaluate the nbTMB (a mutation burden) in terms of its ability to inform on the survival heterogeneity among patients with high TMB undergoing immunotherapy, and to investigate whether it could serve as a differentiating marker for drug sensitivity, particularly in the context of cisplatin treatment in ovarian cancer. Additionally, we aim to assess the potential of mlTNB (a non-B burden) as a marker for survival and outcome risk stratification in early-stage pancreatic cancer.

These aims are based on the hypothesis that a more nuanced approach, incorporating non-B DNA information, can enhance the predictive power of traditional biomarkers and potentially guide the development of targeted therapeutic strategies. Through our investigative lens, we seek to elucidate whether these novel biomarkers can provide a more detailed understanding of tumour behaviour and treatment responses, ultimately contributing to the advancement of precision oncology.

## Results

### The design of nbTMB and mlTNB, based on non-B and mutation co-localisation

Our investigation unveils two novel biomarkers: nbTMB (non-B-informed TMB) and mlTNB (mutation-localised tumour non-B DNA burden), aiming to quantify the multi-dimensions of genomic instability using both tumour mutations and non-B DNA at a sample level.

**Fig. 1:**
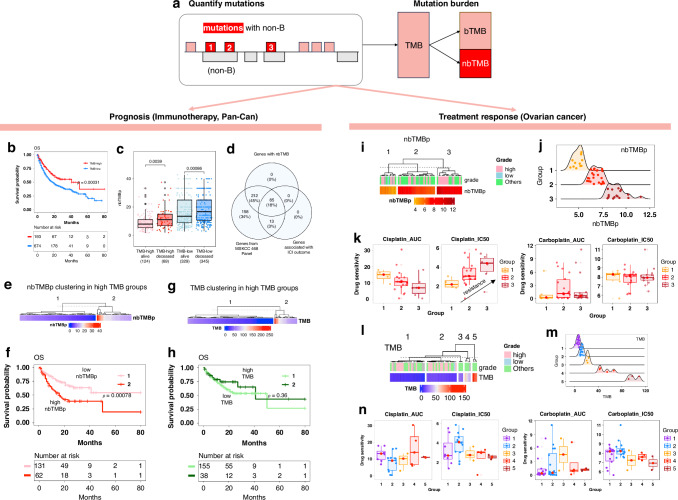
nbTMBp predicts patient outcome and drug sensitivities. **a** Schematic representation of non-B-informed mutation quantification, showing the calculation of nbTMBp and its role as an indicator of TMB composition, bTMB (traditional Tumour Mutation Burden), and nbTMB (non-B-informed Tumour Mutation Burden), emphasising the fraction of mutations associated with non-B DNA structures. **b** Kaplan-Meier survival analysis contrasting overall survival (OS) in patients for Pan-Can patients (*N* = 867) with high vs. low TMB undergoing immunotherapy, highlighting the prognostic significance of TMB levels. The high TMB is define defined as the top 20% within each cancer type. **c** Stratification of pan-cancer patient survival outcomes by TMB levels, with a secondary differentiation based on OS status. Among pan-can patients categorised by TMB levels (high or low), a further distinction into ‘alive’ and ‘deceased’ based on overall survival reveals that the deceased cohort consistently exhibits a higher nbTMBp percentage across both TMB categories. **d** Venn diagram depicting the gene overlap between the MSKCC-Panel-468, genes with non-B DNA mutations and genes linked to immune checkpoint inhibitor (ICI) response, suggesting a potential connection between non-B DNA mutations and immunotherapy effectiveness. **e**, **f** Comparison of survival rates within the high TMB patient subset, showing that higher nbTMBp is associated with decreased survival, thereby offering additional stratification within this group. **g**, **h** Analysis within the high TMB category, indicating that further dissecting patients on TMB exclusively does not significantly stratify patients’ survival outcomes. **i**, **j** Clustering of ovarian cell lines by nbTMBp to identify distinct groups with varying levels of nbTMBp, potentially reflecting differential drug sensitivity. **k** nbTMBp shows a linear trend of increasing drug resistance of Cisplatin. This is evident in both IC50 metrics (where a lower value indicates increased sensitivity) and dose-response AUC (where a higher value indicates increased sensitivity). It demonstrates that increased nbTMBp correlates with heightened resistance to Cisplatin in ovarian cancer cell lines, suggesting the utility of nbTMBp in predicting drug response. Such a correlation is absent in the case of another platinum-based compound Carboplatin. **l** Heatmap and ridge plot illustrating the lack of a clear relationship between TMB alone and drug sensitivity of Cisplatin and Carboplatin in ovarian cancer cell lines, indicating the potential advantage of incorporating nbTMBp for more refined predictions. **m** Distribution of ovarian cancer cell lines across TMB-based clusters, with the colour gradient representing the range of TMB values. **n** Box plots showing drug sensitivity metrics across TMB-defined clusters for Cisplatin and Carboplatin, indicating the absence of a consistent pattern between TMB and drug response, thus underscoring the potential value of nbTMBp in therapeutic decision-making.

First, nbTMB quantifies mutations co-localised within non-B forming regions (Fig. 1a) as a non-B informed tumour mutation burden TMB. The mutation signatures are first extracted from each tumour profile and then mutation sites overlapped with non-B DNA motifs are further quantified at genomic-wide. We also calculate nbTMB percentage (nbTMBp) to describe the proportion of tumour mutations co-localised with non-B DNA motifs, relative to total TMB.

**Fig. 2:**
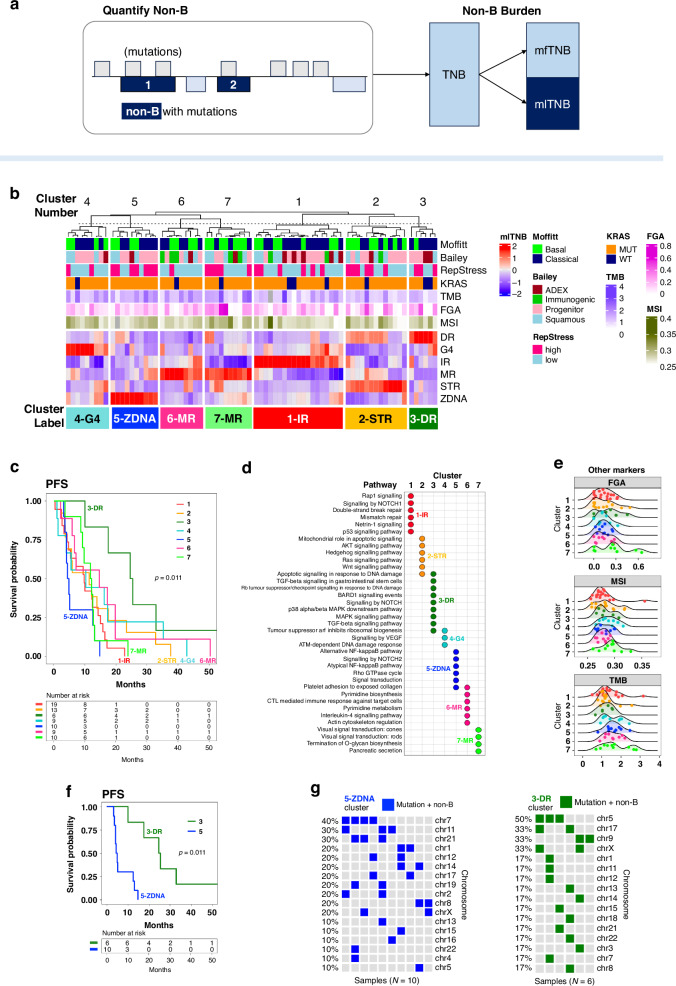
mlTNB predicts prognosis in early-stage pancreatic cancer. **a** Diagram delineating the categorisation of tumour non-B DNA burdens: total non-B burden (TNB), mutation-localised tumour non-B burden (mlTNB) and mutation-free tumour non-B burden (mfTNB). **b** Clustering analysis of early-stage pancreatic cancer patients with progression, resulting in seven distinct patient sample clusters. Each group is characterised by unique mlTNB loads, which are differentiated by the type of non-B DNA motifs present, such as direct repeats (DR), G-quadruplexes (G4), inverted repeats (IR), mirror repeats (MR), short tandem repeats (STR) and Z-DNA (ZDNA). **c** Kaplan-Meier plots illustrating progression-free survival for the seven patient clusters identified, providing insight into the prognostic value of mlTNB categorisation. **d** Summary of pathway enrichment analysis, which underscores the predominant gene mutation signatures associated with each of the mlTNB clusters, potentially linking molecular pathways with patient prognosis. **e** Comparative overview of the distribution of fractional genome alteration (FGA), TMB and microsatellite instability (as measured by MANTIS score) across the clusters, indicating a lack of significant variation among the groups, which all show low TMB (median TMB < 2), low FGA (median < 30%) and MSS (median MANTIS score < 0.3), underscoring mlTNB as a driving factor stratifying the patients. **f** Comparison of PFS between the cluster with the most extended median PFS (mlTNB-DR, cluster 3) and the cluster with the shortest median PFS (mlTNB-ZDNA, cluster 5), emphasising the differential impact of non-B DNA motif types on patient outcomes. **g** Visual representation of the chromosomal distribution of non-B DNA and mutation co-localisations contributing to the mlTNB burden across different chromosomes. The x-axis represents samples, and the y-axis represents chromosomes. Each column corresponds to a sample, and each coloured grid indicates a mutation/non-B DNA co-localisation within a specific chromosome for that sample.

Second, mlTNB refers to mutation-localised tumour non-B DNA burden as a quantification of non-B DNAs. Different from nbTMB, the marker, mlTNB, focuses on the counts of non-B motifs that contain mutation sites (Fig. 2a). Due the various non-B types, the mlTNB is further calculated by non-B motif types. For the comparison across non-B types and across samples, the burden value will also be normalised by both the number of mutations and the motif library size.

The two markers are calculated for each tumour profile at sample level. The mutation signatures are extracted from each tumour profile. The genomic-wide non-B forming region are further overlapped with the mutated regions for each tumour profile. Using the overlapped region by counting separately the number of mutation and non-B motifs involved, we are able to derive the two metrics, nbTMB and mlTNB, as the new biomarker to reflect the interplay between mutation and non-B DNA. The metric was further refined by optional normalisations to predict patient prognosis and treatment responses.

In this study, nbTMBp was applied to immunotherapy treatment in ovarian cancer due to the high heterogeneity observed in patient responses within the high-TMB group. nbTMBp refines TMB by quantifying the percentage of mutations co-localised with non-B DNA, providing additional stratification. Conversely, mlTNB was developed for pancreatic cancer, an ‘immune-cold’ cancer with typically low TMB. mlTNB focuses on non-B DNA burden, accounting for the impact of non-B DNA structures on genomic instability.

### nbTMB differentiates survival among TMB-High patient post-immunotherapy

We first describe a Pan-Can immunotherapy analyses in which nbTMBp appears linked with prognosis. High TMB has been reported to be associated with improved immunotherapy response [16–18]. However, within TMB-high patients, outcomes remain heterogenous. Herein, we explore the heterogeneity with TMB high/low groups using nbTMBp to investigate its role as a biomarker associated with post-immunotherapy survival.

We analysed the mutation data from patients who underwent immunotherapy based on the MSK-IMPACT study from 11 different cancer types [17]. Within each caner type, using the 80th percentile of TMB, we assigned patients into TMB -high and -low groups and compared their overall survival (OS) (Fig. 1b). We further stratified patients within each of TMB-High and -Low groups based on their status—alive/censored or deceased. We defined nbTMB for each patient sample by quantifying the numbers of mutations co-localised with non-B forming regions [19]. When comparing median nbTMBp across groups, the TMB-high group had a lower nbTMBp overall, relative to the TMB-low group (Fig. 1c). Within each TMB classified group, median nbTMBp was significantly higher in deceased patients, irrespective of their high/low status (Fig. 1c). A gene-level analysis of immune response signatures [20] revealed an 86% overlap between mutations co-localised with non-B motifs and immune checkpoint inhibitor-outcome-linked genes (*n* = 98) (Fig. 1d).

Next, we performed clustering on nbTMBp within the TMB-High patients, which revealed two patient subgroups (Fig. 1e), one with median nbTMPp of around 10% that was associated with significantly (*p* < 0.01) shorter OS as compared to TMB-High patients with less than 10% nbTMBp (Fig. 1f). For comparison, the same analysis was applied to TMB in which no significant OS difference was observed (Fig. 1g–h). Altogether, our findings lend support for the further study of nbTMBp as a potential marker of differential survival within TMB-high patients on immunotherapy. These results may reflect the potential contribution from non-B DNA genomic instability in some patients that have poor survival, despite having high TMB.

### Increasing nbTMB is associated with decreased cisplatin sensitivity in ovarian cancer

We next explore the use of nbTMBp as a marker of altered cisplatin drug sensitivity in ovarian cancer. Cisplatin resistance is a major hurdle in effectively treating ovarian cancer [21]. Although cisplatin is commonly used for ovarian cancer treatment, drug resistance often arises due to a faulty apoptotic process, reducing treatment effectiveness [22–26]. Among the 57 ovarian cell lines with mutation profiles [27], ~40% have TMB greater than ten and a median fraction of genome altered (FGA) of ~50%, which supports the potential role of genomic instability in treatment resistance. Investigating how cells signal in response to chemotherapy from the non-B DNA perspective of genomic instability may shed light on treatment outcomes.

We defined an ovarian cell line specific mutation signature and corresponding nbTMBp for use in a cluster analysis that identified three cell line groups of varying (low to high) nbTMBp (Fig. 1i, j). Median nbTMBp significantly differed among the three clustered cell line groups. Tests of association between TMB, FGA and tumour grade with nbTMBp-derived cell line clusters lacked significance, as did a correlation between TMB and nbTMBp among the ovarian cell lines. We examined the effect of clusters on cisplatin drug sensitivity in which increasing nbTMBp was significantly associated with decreasing cisplatin sensitivity. This finding was consistent for dose-response AUC with cisplatin (Fig. 1k). For comparison, we performed the same analyses on carboplatin sensitivity which did not show the same result, suggesting a cisplatin specific nbTMBp effect. Additionally, the same cluster analysis on TMB failed to show a similar result (Fig. 1l, m). Altogether, our findings show support for the further exploration of nbTMBp as a potential marker of cisplatin sensitivity that may help to explain resistance when all other markers indicate otherwise.

### mlTNB differentiates survival among early-stage pancreatic patient progressors

In contrast to nbTMBp, we next explore the use of mlTNB to quantify non-B burden and its association with survival in pancreatic adenocarcinoma (PAAD). PAAD is a highly aggressive cancer with poor outcome. Existing genomic instability measures have not proven informative in differentiating survival into clinically translatable patient groups for risk stratification [28]. As opposed to focusing on mutation numbers, mlTNB quantifies non-B DNA regions that contain mutation sites which can be classified according to non-B structure types to provide a more nuanced perspective [7] (Fig. 2a).

Using the mutation profiles of 76 TCGA early-stage pancreatic patients who progressed, we quantified mlTNB for each sample and used it in a cluster analysis resulting in seven patient groups with differentiated non-B structure types (Fig. 2b) that significantly differed in progression-free survival (PFS) (Fig. 2c). Patients with high mlTNB characterised mainly by direct repeat regions (high mlTNB-DR burden) was associated with the longest PFS (*n* = 7, median = 25 months), while patients with high mlTNB in Z-DNA regions had the shortest (*n* = 10, median = 5 months). PFS among other groups were similar: the patient group with high mlTNB from short tandem repeat regions (STR) (*n* = 13, median = 11 months); the sample group with mlTNB from mirror repeats (MR) without inverted repeats (IR) (*n* = 7, median = 12 months); and the group with MR with IR (*n* = 9, median = 15 months).

According to the pathway analysis on gene signatures of sample clusters (see *Methods*), the high mlTNB-DR burden patients had mutation signatures enriched in MAPK and Notch signalling pathways, as compared to the other clusters enriched with double-stranded break and mismatch repair (cluster 1-IR), hedgehog and WNT signalling (cluster 2-STR) and interleukin-4 signalling (cluster 6-MR) pathways (Fig. 2d). There was a lack of significant association between mlTNB clusters with age, race, sex, PAAD subtypes [29, 30], KRAS mutation status and tumour purity. Additionally, there was no significant association between the non-B DNA clusters with markers of genomic instability, TMB, FGA, and MSI-score (measured by MANIS score) [31], whose distributions were similar across clusters and on average, were low in value (Fig. 2e). Specifically, the median TMB was 1.43, the median FGA, 0.13 and the median MSI-score was 0.28 across all groups. In the shortest PFS (high mlTNB-ZDNA, cluster 5-ZDNA) (Fig. 2f), chromosome 7 had the highest prevalence of non-B mutation co-localisation, while the longest PFS (high mlTNB-DR, cluster 3-DR) patient group non-B mutation co-localisation resided mainly on chromosome 5 (Fig. 2g). Our results lend support to the further study of mlTNB as a differentiating marker of survival in PAAD patients that may further inform on their heterogeneous response to treatment.

## Discussion

We explored the integration of non-B DNA information within mutation burden in cancer by the introduction of two makers: nbTMB and mlTNB. Our analysis demonstrates the distinct applications of nbTMBp and mlTNB in different cancer contexts. While nbTMBp enhances the predictive power of TMB in immunotherapy response, mlTNB provides valuable insights into survival outcomes in pancreatic cancer by emphasising non-B DNA burden. The results show the added value from inclusion of non-B DNA data, lending support to the multi-dimension roles of genomic instability arising from non-B structures and their potential impact on cancer prognosis and treatment efficacy.

### Innovation

A highlight of this study revolves around the predictive potential of these biomarkers. For instance, the differentiating capacity of nbTMBp in determining immunotherapy response underscores the importance of decomposing mutation burden into multiple features, such as non-B DNA. Similarly, the insights derived from the ovarian cancer case, where an increasing nbTMBp revealed heightened cisplatin sensitivity, highlights the potential treatment associated response. On the other hand, non-B-specific structures associated with mlTNB presents prediction capability in patient outcome in early-stage pancreatic cancer. Within our discussion, we have underscored the promising potential of integrating non-B DNA information with genomic variant assessments. This integration represents a significant step towards a more sophisticated understanding of tumour genomics and may offer a valuable complement to existing TMB refinements, such as analyses focused on mutations exclusively. Our approach rests on the hypothesis that the genomic landscape, which is intricately shaped by mutation load coupled with the presence of non-B DNA structures, can provide profound insights into tumour behaviour and responsiveness to treatment. The data presented herein suggests that our biomarkers, nbTMB and mlTNB, by capturing the complexity of the tumour genome—beyond simple mutation counts—have the potential to convey unique prognostic information. Such information may remain concealed if the assessment is limited to mutation loads alone. While our study did not perform a direct comparison with every variant of TMB enhancement, the initial findings advocate for the value of our biomarkers and set the stage for subsequent comparative analyses. These future studies will be crucial in delineating the relative advantages of different TMB assessment strategies, furthering our collective goal of refining precision oncology tools.

### Mechanism research

While we demonstrated the patient grouping with clustering analyses, optimising the thresholds for biomarkers with continuous value in clinical application remains a challenge. While the correlation between nbTMB/mlTNB and treatment responses is compelling, the mechanism remains unexplored. Future prospective studies are essential to elucidate the mechanisms underlying the observed associations as well as to validate the clinical applicability of these biomarkers. The potential hypothesis includes that the frequency and specific localisation of mutagenesis at non-B DNA motifs could influence genomic stability and, consequently, tumour behaviour [10]. Additionally, the interplay between tandem repeats, microsatellite instability and DNA repair processes could elucidate the propensity for cancer development and progression [32]. Future research, building on our findings, should aim to dissect these complex interactions. For instance, studies could focus on characterising the mutational landscape in the vicinity of non-B DNA structures and investigating the biological consequences of such mutations. Understanding the impact of non-B DNA on the functionality of DNA repair pathways could unveil new therapeutic targets. We explored the potential of nbTMBp as a marker among patients with high-TMB who received immunotherapy across various cancer types. Trends were observed in specific cancer types, such as Glioma and Urothelial cancer, where a higher proportion of patients with elevated nbTMBp were found to have a deceased status. But future studies with larger sample sizes will be needed to validate these preliminary observations. Furthermore, longitudinal studies tracking the evolution of these genomic features in response to treatment could shed light on their role in treatment resistance and disease recurrence. While our study focused on the co-localisation between non-B DNA motifs and mutations for understanding the interactions that influence cancer progression and treatment response, the inclusion of non-B DNA motifs without mutations as a genomic control is conceptually important. Future work will explore genomic regions containing non-B DNA structures without mutations to provide a broader context and validate our findings further. It is our hope that this initial exploration serves as a catalyst for more detailed investigations into the biological underpinnings of non-B DNA-related genomic instability. Such work is essential not only for the advancement of fundamental cancer biology but also for the translation of these genomic insights into practical, clinical interventions that can significantly improve patient outcomes.

### Clinical utility

While our initial findings are promising, we recognise the need for a comprehensive evaluation in a more diverse array of cancers and demographic backgrounds to solidify the biomarkers’ universal relevance. Future studies should aim to encompass a broader dataset, particularly those inclusive of varied immunotherapy responses, to determine the robustness of our biomarkers across different genomic landscapes and treatment regimens. Such expansion is crucial not only for validating the efficacy of nbTMB and mlTNB but also for exploring their prognostic capabilities across a wider patient demographic.

For the clinical workflow integration, transitioning from research to real-world clinical application, the integration of nbTMB into clinical workflows raises important considerations. The detection of non-B regions requires genome-wide sequencing capabilities, which are becoming increasingly available in clinical settings. However, the implementation of such comprehensive testing necessitates the development of streamlined protocols and cost-effective methodologies. One potential avenue to explore is the creation of panel-wise nbTMB, which would parallel the current clinical approach to TMB, allowing for a more targeted and economical application while retaining the enriched information provided by non-B DNA contexts. The logistics of incorporating these biomarkers into standard care must be addressed to ensure their practicality and accessibility for clinical use.

In summary, we present two cancer biomarkers of non-B DNA associated burden that, with the role of non-B DNA in genomic instability, offers a novel expansion over existing markers for potentially explaining heterogenous outcomes and treatment responses in cancer.

## Methods

### The calculation of nbTMB

#### nbTMB (non-B co-localisation tumour mutation burden)

nbTMB focuses on mutations, particularly those co-localised with non-B regions. The steps to calculate this metric include: (1) identify mutation signature within the dataset for each sample; (2) examine each mutation sites to determine whether it is co-localised with non-B motifs; (3) count all mutations that fall within these regions to derive the nbTMB value. As a derived metric, nbTMB Percentage (nbTMBp) provides a normalised perspective of nbTMB in relation to the total TMB to quantify the information complexity of the TMB. It is calculated using the formula:

$${nbTMB\; percentage}({nbTMBp})=\frac{{nbTMB}}{{TMB}}$$

Where:

nbTMB = Number of mutations co-localised with non-B regions.

TMB = Total tumour mutation burden for a given sample.

### The calculation of mlTNB

#### mlTNB (mutation co-localised non-B Burden)

mlTNB is a metric that quantifies the burden of mutations co-localised with non-B motifs. To calculate mlTNB (mutation-localised tumour non-B Burden), we followed these steps: (1) Identify the mutation signature for each sample, including SNVs and indels. (2) Examine the genomic positions of non-B DNA motifs and determine if they overlap with mutation sites. (3) Count all non-B DNA motifs containing at least one mutation to derive the mlTNB value.

We chose a cutoff of one mutation to be inclusive and retain more signals, given the average length of non-B DNA (~25 bases). This decision ensures comprehensive capture of significant mutations within non-B DNA motifs.

For extension, considering there are multiple type non-B type such as G4, H-DNA, Z-DNA and so on, mlTNB can be derived for each of the non-B type specifically. Additionally, to enable comparison between samples and non-B types, the mlTNB is further normalised by the mutation size factor (divide the counts by total mutation length in each sample) and the non-B library factor (divide the counts by the total size of each non-B type). The calculation is described below:

$${mlTNB}=\frac{{counts\; of\; nonB\; motifs\; overlapped\; with\; mutation\; sites}\,}{{Total\; nonB\; library\; size}\times {Total\; length\; of\; mutation\; sites}\,}$$

Where:

mlTNB = mutation-localised tumour non-B burden

The mutation co-localised non-B Burden is calculated using the ‘burden in batch’ function from the web server NBBC (Non-B DNA Burden in Cancer) [7].

### Mutation signatures for CCLE and TCGA

The mutation data was extracted from two major repositories: the Cancer Cell Line Encyclopaedia (CCLE) and The Cancer Genome Atlas (TCGA) [33]. Both sources offer a comprehensive view of mutational landscapes. The mutation data for patient samples and cell lines are separately downloaded from UCSC Xena [34]. The specific dataset used for our analysis was identified as CCLE_DepMap_18Q2_maf_20180502. It contains somatic mutations include Single Nucleotide Polymorphisms and small INDELs (insertions/deletions). Mutational calls have been merged, focusing on the coding region and filtering out germline mutations. For patient mutation data, the somatic mutation dataset from TCGA is labelled as ‘mc3.v0.2.8.PUBLIC.maf.gz’ [35]. The genome assembly for both datasets is hg19 build. Early-stage pancreatic cancer TCGA patients were defined by Stage I or II pancreatic adenocarcinoma with a predicted tumour purity [36] of at least 25% and no history of neuroendocrine diagnoses, prior treatments, or non-pancreatic origins [37].

### Non-B forming motifs data source

The data for non-B motifs was originally sourced from the Non-B DB 2.0 database [38] and downloaded the NBBC (non-B burden in cancer) web server [7]. The dataset comprises seven non-B structures: A-phased repeats (*n* = 2386), G-quadruplex motifs (*n* = 361,232), Z-DNA motifs (*n* = 404,192), inverted repeats (*n* = 5,771,570), mirror repeats (*n* = 1,378,864), direct repeats (*n* = 1,113,354) and short tandem repeats (*n* = 2,826,360).

### Genomic and survival data for Immunotherapy Patients

The Pan-Can immunotherapy data was sourced from processed mutation and clinical data using the Memorial Sloan Kettering Cancer Center (MSKCC) project available through cBioPortal [39]. This dataset includes genomic and survival information from 1,661 tumour-normal pairs, covering a diverse range of cancer types. All samples within this collection were sequenced using the MSK-IMPACT assay.

### Clustering analysis

The K-means clustering was performed using the baseR kmeans function. The visualisation of clustering results in heatmaps are further conduct by the ComplexHeatmap package [40]. The K-means algorithm is sensitive to the magnitude of the data, and variables with larger scales can dominate the clustering results. Before the application of the K-means algorithm, the dataset was standardised to ensure that each feature contributed equally to the clustering process. The optimal number of clusters is determined be consensus clustering and evaluation methods including the elbow method and silhouette scores [41].

### Pathway analysis on gene signatures of sample clusters

While calculating the mlTNB for each tumour sample, we identified mutations that colocalize with each type of non-B DNA motif. Based on these mutations, we extracted the genes where these mutations are located and formed a signature for each non-B DNA type in each sample. For each patient cluster containing several samples, we created a union of the genes from each sample’s signature to form a gene set for each cluster. This gene set was then used to perform pathway analysis for each cluster.

### Drug sensitivity and survival comparison

The drug sensitivity data was obtained from the CREAMMIST database [42]. The survival data for TCGA was downloaded from cBioportal under TGCA PanCan Altlas Studies [39, 43]. The boxplot visualisations are generated with ggplots [44] and ggpubr [45] package. The survival analysis is conduct by survival and ggsurvfit R packages.

### The definition of high-TMB status and the potential use of nbTMB in high-TMB status

The determination of high tumour mutation burden (high-TMB) status is adapted to be cancer type-specific rather than a fixed universal threshold. Recognising the variability of TMB benchmarks across different cancer types, high-TMB is defined as the top 20th percentile within each cancer type’s TMB distribution. This percentile-based approach ensures that the high-TMB classification is relevant and reflective of the mutational landscape inherent to each cancer type.

The potential use of non-B-informed tumour mutation burden (nbTMB) in high-TMB status serves as a novel and refined stratification method. Within the cohort of patients classified as high-TMB by this relative definition, nbTMB provides an additional layer of distinction by quantifying the proportion of mutations co-localised with non-B DNA motifs. This biomarker is designed to identify subgroups of high-TMB patients who may have different clinical outcomes due to the influence of non-B DNA regions on genomic stability and treatment response. Through this methodology, we aim to explore the prognostic implications of nbTMB in high-TMB patients across various cancer types.

## Data Availability

The immunogenomic dataset derived from Samstein et al.’s cohort are available at https://www.cbioportal.org/study/summary?id=tmb_mskcc_2018. The scripts for analyses in this study is available under a GPL-3.0 license in the Kowalski Lab GitHub repository (https://github.com/kmlabdms/MLNB).
